# The Pathophysiological Mechanisms and the Quest for Biomarkers in Psoriasis, a Stress-Related Skin Disease

**DOI:** 10.1155/2018/5823684

**Published:** 2018-01-28

**Authors:** Mircea Tampa, Maria-Isabela Sarbu, Madalina-Irina Mitran, Cristina-Iulia Mitran, Clara Matei, Simona-Roxana Georgescu

**Affiliations:** ^1^“Carol Davila” University of Medicine and Pharmacy, Bucharest, Romania; ^2^Department of Dermatology, “Dr. Victor Babes” Hospital, Bucharest, Romania

## Abstract

Psoriasis is a physically, emotionally, and socially invalidating multifactorial disorder, with a significant impact on the patients' quality of life. Stress is one of the leading triggers for psoriasis and has been associated with disease onset and subsequent flare-ups, while the flare-ups by themselves often lead to psychological discomfort. The treatment of psoriasis is individualized, depending on the patients' measurable severity of illness, as well as the impact the skin condition has on patients' quality of life, as assessed by standardized questionnaires. The clinical scales used nowadays for measuring the severity of psoriasis are characterized by low reproducibility and high variability between examiners. Hence, there is a real need to identify objectively measurable biomarkers to standardize the assessment of the severity of psoriasis. We aim to review the pathophysiological mechanisms involved in psoriasis, focusing on the most critical advances in psoriasis biomarker discovery, pointing out those biomarkers which have also been studied in other stress-related conditions, thus emphasizing the relationship between psoriasis and stress.

## 1. Introduction

Psoriasis is a chronic, immune-mediated, polygenic skin disease with a universal occurrence affecting approximately 2% of the Caucasian population [1, 2]. It is a physically, emotionally, and socially invalidating condition with a great impact on the patients' quality of life [3]. Patients with psoriasis often experience social stigma. Psoriasis can appear at any age, but two peaks in age onset have been reported: the first between 20 and 30 years and the second between 50 and 60 years [4]. Males and females are equally affected. Several clinical variants of disease have been described, namely, guttate psoriasis, erythrodermic psoriasis, and pustular psoriasis, with chronic plaque psoriasis vulgaris accounting for more than 90% of cases [2–4].

Psoriasis may not be confined to the skin only; the joints may also be involved; psoriatic arthritis affects 5–30% of patients with cutaneous disease; both axial and peripheral joints can be involved [3]. In most cases, psoriatic arthritis appears at least one decade after the skin disease while in the rest the two disorders occur simultaneously or the arthropathy precedes the cutaneous disorder [2]. The most important risk factors for developing psoriatic arthritis are female gender, early onset, genetic predisposition, and early detection of radiographic signs [4].

Skin diseases are a leading cause of nonfatal disease burden [5], and psoriasis is one of the main dermatological disorders associated with psychological distress [6]. Studies showed that psoriatic patients have an increased risk of psychiatric comorbidities and suicidal ideation compared to patients suffering from other dermatological diseases such as melanoma and allergic disorders [7, 8]. Moreover, it was shown that the quality of life of psoriatic patients is as deteriorated as that of patients suffering from cardiovascular disease or cancer [9]. Patients with psoriasis often feel stigmatized, depressed, or anxious. Sexual impairment is more important in the more severely affected patients, and alcoholism is more frequent in psoriatic patients than in general population [10–15]. There is also evidence that psoriatic arthritis is associated with depression and suicidal behavior [13].

Stress is one of the best-known triggers for psoriasis. It has been associated with disease onset, flare-ups, and psychological distress [16]. Anxiety might occur in psoriatic patients due to disfigurement, stigmatization, or chronic pruritus. Disfigurement, stigmatization, and dissatisfaction with treatment could also lead to depression. On the other hand, psoriasis might be triggered or worsened by psychiatric conditions like depression and anxiety within a vicious cycle [16]. In a recent systematic review, the authors found that, apart from anxiety and depression, psoriasis is linked to many mental disorders like eating disorders, sleep disorders, sexual disorders, substance abuse and dependence, psychoses, bipolar disorder, or somatoform disorders [16].

Given the accumulating evidence that psoriasis might be associated with suicidal ideation [6, 13, 14, 17], the subject captured the interest of several authors. Two recent studies had a paramount importance in demonstrating the relationship between psoriasis and suicidality [18, 19]. In a systematic review and meta-analysis of eighteen studies with 1,767,583 participants, of whom 330,207 were diagnosed with psoriasis, Singh et al. found that patients with psoriasis were more likely to exhibit suicidal behaviors, to attempt suicide, and to complete suicide than patients without psoriasis, patients with a more severe disease and younger patients having the highest risk [18]. A population-based cohort study which comprised 408,663 individuals, including 57,502 patients with mild psoriasis and 11,009 patients with severe psoriasis, also found an increased risk of self-harm and nonfatal suicide attempts in patients with severe psoriasis. According to this study however, the risk of completed suicide was not increased [19].

Psoriatic patients also experience a great amount of psychological stress, and, in 40 to 80% of patients, the disease activity is influenced by stressful events [12]. Some authors showed that two main factors contribute to the psychosocial impact of psoriasis: stress associated with engaging in anticipatory or avoidance coping behavior that is effected to limit the sociocognitive intrusiveness of psoriasis and stress resulting from patients' beliefs or actual experiences of being evaluated by others solely on the basis of their skin [20]. Interestingly, the psychological burden of psoriasis is not always proportional to the disease severity, as measured by clinical scales available so far. For that reason, it is recommended that measures assessing the psychosocial morbidity should also be considered when determining psoriasis severity [11].

The mechanisms through which psychological stress interferes with psoriasis onset or exacerbations are not completely understood. Psychoneuroimmunology studies showed that stressors can affect the immune function [21]. Therefore, stress determines the secretion of corticotropin-releasing hormone (CRH) in the hypothalamus. CRH determines high serum levels of adrenocorticotropic hormone (ACTH) which induces the release of glucocorticoids. CRH is also involved in the secretion of noradrenaline in the peripheral sympathetic nervous system and noradrenaline and adrenaline in the adrenal medulla. Stress therefore increases the peripheral levels of neurohormones. The cells of the immune system, including T lymphocytes, B lymphocytes, and monocytes, express receptors for those hormones. Chronic stress has been associated with chronic elevations in proinflammatory cytokines, especially interleukin- (IL-) 6, tumor necrosis factor- (TNF-) *α*, and IL-1 beta [21–23]. While the role of inflammation in the development of psoriasis is already well established, there is increasing evidence that inflammation might also play a role in the development of neuropsychiatric disorders. Depression has therefore been associated with high levels of IL-6, TNF-*α*, IL-1 beta, C-reactive protein (CRP), chemokines, and adhesion molecules [21]. IL-6 and TNF-*α* alter the metabolism of norepinephrine, serotonin, and dopamine and lead to symptoms of depression. On the other hand, IL-6 promotes the production of T helper (Th) 17 cells and, together with TNF-*α*, plays a central role in the development of psoriasis lesions [16]. The administration of cytokines or cytokine inducers in healthy volunteers and laboratory animals determines symptoms of depression and anxiety which further increase the levels of cytokines. On the other hand, the administration of anti-inflammatory therapies in patients with inflammatory disorders like psoriasis has been associated with significant improvement in the depressive symptoms [24–27].

## 2. The Need for Biomarkers in Psoriasis—beyond the Scales Used to Assess Physical and Psychological Burden of the Disease

The treatment is individualized depending on the patients' measurable severity of illness [2, 4]. The Psoriasis Area and Severity Index (PASI) is nowadays considered the gold standard for evaluating the activity of the disease. This score takes into account the intensity of erythema, lesion thickness, and scaling in the four regions of the body, namely, the head and neck, trunk, upper limbs, and lower limbs, and the percentage area affected. When assessing the severity of the disorder and choosing the treatment, the physician must also consider the patients' perception of the disease [2]. Several tools for quality of life measurement are nowadays available. The Dermatology Life Quality Index (DLQI) is a self-reported questionnaire of ten items which evaluates the impact of the disease on daily activities, feelings, work, studies, personal relationships, and treatment. The DLQI score ranges from 0—no effect on subjects' life—to 30—extremely large effect on subjects' life [28]. Psoriasis-specific measures are also available. The Psoriasis Life Stress Inventory (PLSI) is a 15-item questionnaire which measures the psychosocial stress associated with coping with everyday events [29, 30]. The Psoriasis Disability Index (PDI) consists of 15 psoriasis-specific questions which address the disability in daily activities, employment, personal relationships, leisure, and treatment [29, 31]. Psoriasis Index of Quality of Life (PSORIQoL) is a 25-dichotomous item instrument designed for clinical practice and trials which assesses the capability of individuals to satisfy their needs [29, 32, 33].

The reliability of these scores is uncertain as there is high variability and low reproducibility between physicians. Scientists are therefore trying to identify biomarkers which can be objectively assessed in order to standardize the measuring of the severity of psoriasis [34, 35].

A biomarker is a characteristic that is objectively measured and evaluated as an indicator of normal biological processes, pathogenic processes, or pharmacologic responses to a therapeutic intervention [36]. In clinical practice, they can be used as diagnostic tools, for staging of diseases, as indicators of prognosis, or for monitoring the clinical response after an intervention. They can also help understand the pathogenesis of various diseases or develop novel therapies [36]. In the future, biomarkers might play a central role in personalized therapy as they might help identify patients who will not respond to a certain treatment or who might get adverse reactions [37]. Moreover, biomarkers have had a very important role in understanding the pathogenesis of psoriasis and facilitated the development of biological therapies [38].

## 3. General Overview of Molecular Mechanisms Involved in the Pathogenesis of Psoriasis

Psoriasis is histopathologically characterized by elongated rete ridges, hyperkeratosis with parakeratosis, and dilated vessels in the dermal papillae. These histopathological changes are responsible for the clinical appearance of psoriasis. Therefore, the silvery-white scales are the result of parakeratosis. The lesions are elevated and well demarcated due to the elongation of rete ridges while the erythema is the result of the dilated dermal vessels [2].

**Figure 1:**
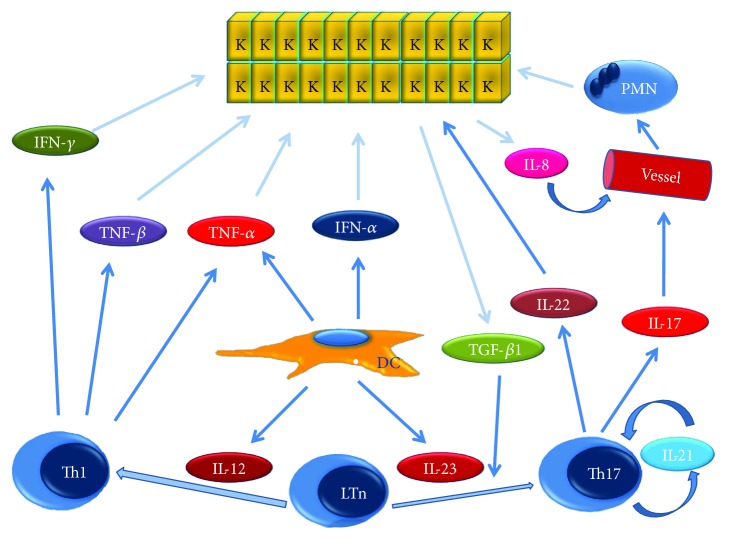
Molecular mechanism in psoriasis—overview. See text for explanation. DC = dendritic cell, IFN-*α* = interferon-*α*, IFN-*γ* = interferon-*γ*, IL-8 = interleukin-8, IL-12 = interleukin-12, IL-17 = interleukin-17, IL-21 = interleukin-21, IL-22 = interleukin-22, IL-23 = interleukin 23, K = keratinocyte, LTn = naïve T lymphocyte, PMN = polymorphonuclears, Th1 = T helper 1, Th17 = T helper 17, TGF-*β*1 = transforming growth factor-beta 1, TNF-*α* = tumor necrosis factor-*α*, and TNF-*β* = tumor necrosis factor-*β*.

Almost all cell types found in the skin are involved in the pathogenesis of psoriasis. Keratinocytes produce IL-8, IL-12, IL-15, IL-18, TNF-*α*, vascular endothelial growth factor (VEGF), and platelet-derived growth factor (PDGF). Dendritic cells produce interferon- (IFN-) *α*, TNF-*α*, IL-20, and IL-23. Th1 lymphocytes secrete IFN-*γ*, TNF-*α*, and TNF-*β*, Th17 lymphocytes produce IL-17, IL-21, and IL-22, and the dermal mesenchymal tissue produces keratinocyte growth factor (KGF); the interaction of all the cytokines and chemokines is extremely complex and results in the amplified proliferation of keratinocytes, angiogenesis associated with the occurrence of dilated capillaries, and the maintenance of inflammation. These processes underlie the occurrence of the psoriasis plaque. A central role in the pathogenetic mechanisms involved in psoriasis is played by TNF-*α* (Figure 1) [39–43].

CD8 T cells play a central role in the pathogenesis of psoriatic arthritis. Those cells are found in high levels in synovial tissue and fluid. Activated T cells release cytokines and chemokines which affect tissues directly and indirectly by activating other inflammatory cells [2, 44].

## 4. Psychological Stress and Psoriasis

Since stress is an important trigger for psoriasis, the mechanisms through which psychological stress might exacerbate the disease were studied by several authors. Proposed theories include altered distribution of leukocyte subsets and cytokine productions in psoriatic patients exposed to stress, consisting in elevated CD4^+^ T cells and monocytes and decreased levels of CD3^+^/CD5^+^ T cells [45], and an inadequate response of the hypothalamic-pituitary-adrenal (HPA) axis [46]. The role of stress in the enhancement of neurogenic inflammation is also discussed as vasoactive intestinal peptide (VIP), and calcitonin gene-related peptide- (CGRP-) immunoreactive nerves were observed in the papillary dermis of lesional skin in stressed patients [46, 47].

Normally, stress activates the central HPA axis and determines the release of stress hormones like CRH and arginine vasopressin in the hypothalamus which determines the release of ACTH in the anterior pituitary. ACTH regulates the secretion of glucocorticoids [46]. In psoriatic patients who are highly reactive to stress, the levels of cortisol are low. As a result, there is increased inflammation and immune overactivity [46, 48, 49].

The association between PASI scores and CRH receptor type 1 (CRH-R1) was studied in 46 adult patients with psoriasis and 20 healthy controls. The expression of CRH-R1 was determined immunohistochemically in skin biopsies. A statistically significant increase of CRH-R1 was found in psoriatic lesions, and a correlation between the severity of the disease as measured by PASI and the expression of CRH-R1 was identified. These results support the role of stress in the exacerbations of the disease [50].

The role of neurotensin (NT), a peptide widely distributed in the central and peripheral nervous systems, which can enhance the ability of CRH to increase skin vascular permeability, has also been studied in patients with psoriasis. High serum levels of NT were identified in psoriatic patients as compared to controls. The expression of genes for NT and NT receptor-1 in psoriatic skin was decreased compared to controls. NT induces the release of VEGF, a molecule involved in the pathogenesis of psoriasis, from mast cells and might partially explain the role of stress in the skin disease [51].

## 5. The Quest for Biomarkers in Psoriasis

Several molecules have been studied as possible biomarkers in psoriasis, the most important being soluble biomarkers, tissue-associated biomarkers, markers of oxidative stress, genetic markers, and cell subsets (Table 1).

### 5.1. Soluble Biomarkers

#### 5.1.1. C-Reactive Protein and Other Nonspecific Indicators of Inflammation

Since psoriasis is a chronic inflammatory disease, Rocha-Pereira et al. evaluated the extent of inflammatory response in mild and severe psoriasis by measuring the levels of CRP, fibrinogen, erythrocyte sedimentation rate (ESR), haptoglobin, and C3 and C4 complement proteins. The authors showed that all the patients with psoriasis had higher levels of CRP than controls. 97% of the patients with psoriasis also had higher levels of haptoglobin than the controls. Fibrinogen, ESR, and C3 and C4 complement proteins were also higher but not to the same extent. In this study, inflammatory markers were significantly lower in patients with inactive psoriasis than in patients with active psoriasis [52]. Coimbra et al. studied CRP as a potential monitor for psoriasis vulgaris. The authors found that CRP levels correlate with PASI and concluded that CRP could be used to assess the severity of psoriasis and to monitor the response to treatment [53].

CRP levels were also measured in patients with psychological disorders. In a study performed on 80 patients, elevated levels of CRP were associated with depression and anxiety [54]. A study performed on 73,131 participants showed that elevated CRP levels are associated with a high risk of psychological distress and depression [55]. Other authors also found similar results [56].

Platelet P-selectin was also proposed as a possible biomarker for psoriasis. Platelet P-selectin is a cell adhesion molecule of the platelets which, after platelet stimulation, is translocated to the plasma membrane and acts as a receptor for monocytes and neutrophils [57]. Garbaraviciene et al. investigated platelet P-selectin in patients with psoriasis and patients with other inflammatory skin disorders and concluded that the level of platelet P-selectin is increased in patients with psoriasis and it correlates with the severity of psoriasis as measured by PASI. The authors also showed that there is a strong correlation between the P-selectin expressed on platelets and the soluble P-selectin which can be easily measured through routine measures and concluded that plasma P-selectin could be used as a biomarker in psoriasis [58].

High levels of platelet P-selectin and norepinephrine and delayed recovery were also identified in depressive and anxious patients who were exposed to an acute psychological stress task [59].

#### 5.1.2. Vascular Endothelial Growth Factor (VEGF)

VEGF is a proangiogenic factor found in high levels in psoriasis. It is responsible for the increased dermal vascularity which is specific for the psoriatic plaque [35, 60–62]. One study measured the concentrations of VEGF and VEGF-soluble receptors (sVEGF R1 and sVEGF R2) in patients with psoriasis before and after treatment. The scientists discovered that VEGF levels were correlated with the disease activity as measured by PASI and that psoriasis treatment led to a reduction in VEGF serum levels. They also discovered that high levels of sVEGF R1 might be an indicator of clinical improvement [63].

Chen et al. performed a meta-analysis in which they included studies investigating the effect of narrow band UVB therapy on serum levels of VEGF and IL-8 in patients with psoriasis. They included 13 studies with a total of 400 patients with psoriasis and 221 controls. The authors discovered that psoriatic patients had significantly higher levels of VEGF than the healthy controls prior to NB-UVB treatment and that the levels of VEGF significantly decreased after treatment. The levels of serum IL-8 were not significantly different between psoriatic patients and controls before treatment but significantly decreased after NB-UVB treatment. The authors concluded that VEGF and IL-8 are sensitive markers for assessing the response to treatment [64].

VEGF is currently regarded as a possible target for novel therapies for psoriasis as some authors showed that bevacizumab, a monoclonal antibody against VEGF, determined complete remission of psoriasis lesions in a patient treated for colon cancer [65]. Another study performed on a mouse model of psoriasis showed that systemic blockade of VEGF-A by a monoclonal antibody determined normalization of epidermal proliferation, decreased the size of dermal vessels, and reduced inflammatory cells [61, 66]. Further research is however necessary.

The role of VEGF and brain-derived neurotrophic factor (BDNF) in the pathogenesis of mental disorders was also studied. VEGF and BDNF are involved in neurogenesis and synaptic plasticity. Long-term stress determines permanent changes in the brain structures. Animal studies showed that stress is associated with low levels of BDNF and VEGF in the brain and with behaviors characteristic for depression [67].

#### 5.1.3. Transforming Growth Factor- (TGF-) *β*

Dysregulation of TGF-*β*, especially the TGF-*β*1 isoform, was reported in patients with psoriasis. Overexpression of TGF-*β*1 in keratinocytes induces inflammation in several skin diseases [68]. One study evaluated the association between TGF-*β*1 and TGF-*β*2 in scales and plasma of psoriatic patients and disease activity. The authors found a correlation between plasma TGF-*β*1 and disease severity as measured by PASI [69]. The same group of authors also measured the concentrations of TGF-*β*1 and TGF-*β*2 in psoriatic patients before and after treatment to evaluate if those could be indicators of treatment efficacy. The scientists found a correlation between TGF-*β*1 plasma concentrations and disease activity in patients with severe psoriasis (PASI ≥ 15) and concluded that TGF-*β*1 should be considered a biomarker of disease activity during treatment [70]. Other authors also found higher levels of TGF-*β*1 in the plasma of psoriatic patients than in controls and a correlation with the extent of the disease [71]. Meki et al. analyzed the correlation between serum VEGF, TGF-*β*1, and nitric oxide and disease severity in patients with psoriasis and found that they could all be recognized as markers of psoriasis severity [72]. Some authors therefore consider that plasma TGF-*β*1, tissue inhibitors of metalloproteinases- (TIMP-) 1, matrix metalloproteinase- (MMP-) 1, and IL-18 should all be measured in psoriatic patients for superior results [73].

Other authors studied the association between cytokines and depression in an animal model and found that rats exposed to chronic mild stress for four weeks showed depression-like behavioral changes and altered expression of cytokines in the brain, with elevated levels of proinflammatory cytokines (IL-1*β*, TNF-*α*, and IL-6) and decreased levels of TGF-*β* and IL-10 [74].

#### 5.1.4. Antimicrobial Peptides: Human Beta Defensin 2 and Cathelicidin

Antimicrobial peptides play an important role not only in killing pathogenic microorganisms but also in modifying host inflammatory responses. Human beta defensin 2 (HBD-2) and cathelicidin (LL-37) are both increased in psoriasis in response to proinflammatory and type I cytokines and are probably responsible for the low rate of infection in psoriatic skin [2, 75, 76]. It is also believed that they might play a role in the pathogenesis of psoriasis. Studies showed that high HBD-2 genomic copy number is associated with an increased risk of psoriasis [77]. Jansen et al. showed that HBD-2 serum levels correlate with the severity of the disease in patients with psoriasis and concluded that HBD-2 could be a useful biomarker for disease activity [78]. Similar results were found by Jin et al. who analyzed the correlation between HBD-2 and disease activity in psoriatic patients treated with Janus kinase (JAK) inhibitor and concluded that HBD-2 might be a biomarker for monitoring response to treatment [79]. Other authors found that HBD-2 levels are well correlated with IL-17A levels and that HBD-2 could therefore be validated as a biomarker for IL-17A-driven skin pathology [80]. The role of neutrophil extracellular traps (NETs) in inducing HBD-2 production in psoriatic plaques is also studied [81].

Psychological stress was associated with low levels of epidermal antimicrobial peptide expression in mice and an increased risk of developing cutaneous infections [82]. The expression of antimicrobial peptide is regulated by glucocorticoid and *β*-adrenergic mechanisms [83].

#### 5.1.5. S-100 Proteins

S-100 proteins are a large family of dimeric low molecular weight proteins characterized by the presence of two calcium binding sites. They have proinflammatory, antimicrobial, and chemotactic activity. S100A7 (psoriasin), S100A8 (calgranulin A), S100A9 (calgranulin B), and S100A12 (calgranulin C) are markedly increased in psoriasis lesions [2, 75]. Several scientists investigated these proteins to discover if they could be reliable biomarkers in psoriasis. Benoit et al. measured the levels of S100A8 and 100A9 in patients with psoriasis and healthy controls and found significantly higher concentrations of S100A8 and S100A9 in the serum of psoriatic patients. The levels were higher in patients with a more severe disease (PASI ≥ 15) [84]. Anderson et al. measured the serum levels of S100A7 and psoriasin-specific autoantibodies in patients with psoriasis vulgaris. The authors found that even though S100A7 was overexpressed in psoriatic skin lesions, the serum levels of S100A7 were decreased. The psoriasin-specific autoantibodies were also not significantly elevated. Therefore, the authors concluded that S100A7 is not a promising serum biomarker for psoriasis [85]. This result however was not found by other authors. Wilsmann-Theis et al. analyzed the expression of S100A7, S100A8, S100A9, and S100A12 in the skin and serum of psoriatic patients and compared it to those found in patients with atopic dermatitis and lichen ruber and in healthy controls. The authors found that all the studied proteins were highly expressed in the serum and skin of patients with active psoriasis. S100A7 and S100A12 serum levels were closely associated with disease activity in psoriatic patients, and S100A8, S100A9, and S100A12 serum levels decreased after treatment with etanercept. The authors concluded that S100A12 levels are correlated with disease activity and therapeutic response and it is the most significant marker among the S100 proteins [86].

#### 5.1.6. Cytokines

Cytokines have a very important role in the pathogenesis of psoriasis, especially those produced by Th1 cells (IFN-*γ*, IL-2, and TNF-*α*) and those produced by dendritic cells (IL-18, IL-20, TNF-*α*, and IL-23). All those cytokines are potential biomarkers for psoriasis [2, 35].

Arican et al. measured the serum levels of IFN-*γ*, TNF-*α*, IL-6, IL-8, IL-12, IL-17, and IL-18 in patients with psoriasis and in healthy controls. The authors found significantly higher levels of IFN-*γ*, TNF-*α*, IL-6, IL-8, IL-12, and IL-18 in patients with psoriasis. IL-17 levels were slightly higher in psoriatic patients, but the difference was not statistically significant. The authors also found a correlation between IFN-*γ*, IL-12, and IL-18 and the clinical severity and activity of the disease as measured by PASI [87]. Pietrzak et al. measured the serum levels of IL-18 in patients with psoriasis and in healthy subjects and found elevated levels of IL-18 in psoriatic patients as well as a correlation between this cytokine and the disease activity [88]. Similar results were obtained by Flisiak et al. Furthermore, the authors discovered that the combined measurement of IL-18 and TGF-*β*1 could be a biomarker of psoriasis activity [89].

High cytokine levels were also identified in stress-related disorders. A study performed on 38 medical students showed that psychological stress was associated with elevated TNF-*α*, IL-6, IL-1 receptor antagonist (IL-1Ra), IFN-*γ*, and IL-10. High levels of TNF-*α*, IL-6, IFN-*γ*, and IL-1Ra were identified in those students who perceived stress as high when exposed to the stressful event, and high levels of IFN-*γ* and low levels of IL-10 and IL-4 were identified in those with a high anxiety response [23]. Other studies found that acute stress is associated with increased levels of IL-6 and IL-1*β* [22].

Cytokine levels were also measured in psoriatic patients exposed to psychological stress. Mastrolonardo et al. measured the salivary levels of IL-1*β* in 25 patients with psoriasis and 50 controls who were exposed to a standardized stressful procedure. The authors found that, at baseline, mean IL-1*β* levels were higher in patients with psoriasis than in controls. After the stressful event, the level of IL-1*β* increased in the control group but not in the psoriasis group. The scientists concluded that psoriatic patients might have a defective response of the immune system to adrenergic stimuli [90]. A study performed on mice with psoriasis evaluated the influence of sleep loss on inflammatory cytokine levels. The authors found increased levels of proinflammatory cytokines IL-1*β*, IL-6, and IL-12 and decreased levels in the anti-inflammatory cytokine IL-10 and concluded that sleep loss is associated with disease exacerbations [91].

#### 5.1.7. Squamous Cell Carcinoma Antigen 2

The squamous cell carcinoma antigen (SCCA) is a member of the family of inhibitory serine protease inhibitors and serves as a serological marker for advanced squamous cell carcinomas of the cervix, lung, head and neck, vulva, and esophagus. Elevated levels of SCCA are however also identified in inflammatory disorders like psoriasis and atopic dermatitis [92–94]. Two SCCA proteins have been identified: SCCA1 and SCCA2. SCCA2 inhibits the chymotrypsin-like proteinases cathepsin G and mast cell chymase [92].

In a prospective cross-sectional study, performed on 123 patients with psoriasis and 25 healthy controls, serum levels of SCCA2 were measured and compared with PASI. Significantly higher levels of SCCA2 were found in psoriatic patients than in healthy controls, elevated levels of SCCA2 being associated with disease severity. SCCA2 expression in the skin was also assessed using immunohistochemical analysis, and the authors reported increased expression of SCCA2 in lesional skin compared with nonlesional skin of psoriatic patients [95]. Moreover, IL-17 and IL-22 were found to increase SCCA2 production, and the authors therefore concluded that SCCA2 might be a useful biomarker in psoriasis, reflecting T helper 17-type inflammation [95].

### 5.2. Tissue-Associated Biomarkers

#### 5.2.1. Keratins

Keratins are a family of cytoskeletal proteins. They are the major structural proteins of vertebrate epidermis. As keratinocytes migrate from the basal layer to the cornified layer, they express different keratins. Therefore, keratin 5 (K5) and K14 are normally expressed in the basal layer while K1 and K10 are expressed in the differentiating suprabasal layers. K6 and K16 are normally produced in the outer root sheath of hair follicles and nailbed and are not found in the interfollicular epidermis [96, 97]. In psoriasis and other disorders however, K1 and K10, which are markers of terminal differentiation, are replaced by K6 and K16 which are markers of hyperproliferation [98]. IL-1 has an important role in keratinocyte activation and K6 synthesis [97, 99].

Bhawan et al. showed that K16 expression is observed in nonlesional skin from psoriatic patients and concluded that K16 could be a marker of preclinical psoriasis and could help identify people who might develop the disease [100]. Franssen et al. used flow cytometry to identify different epidermal subpopulations. The authors found correlations between mild psoriasis and K10^+^K6^−^ cells and moderate psoriasis and K10^−^K6^+^ cells. They also discovered that the treatment of psoriasis is associated with an increase of the K10^+^K6^−^ cells and a decrease of the K10^−^K6^+^ cells in mild and severe forms of disease [101].

#### 5.2.2. Connexins

Connexins are gap junction proteins. Connexin 30 and connexin 26 are upregulated in psoriatic skin and have been associated with epidermal hyperproliferation [99, 102, 103].

#### 5.2.3. Keratinocyte Hyperproliferation Markers

Bcl-2 family proteins have an important role in apoptosis, some members of this family promoting apoptosis and others inhibiting it. It has been observed that keratinocytes from psoriasis plaques are more resistant to apoptosis than normal keratinocytes. Some members of the Bcl-2 family, especially the proapoptotic proteins Bax and Bak and the antiapoptotic proteins Bcl-2 and Bcl-X, are believed to be involved in the pathogenesis of psoriasis. The increased levels of all these proteins in the skin of psoriatic patients support the idea that they play a role in epidermal hyperplasia [38, 104].

Heat-shock proteins (HSPs) are a large family of proteins which have various important functions, one of those being the protection of cells against apoptosis. HSPs can inhibit the activity of proapoptotic Bcl-2 proteins [105]. Kakeda et al. analyzed the expression of HSP80 in normal appearing skin, psoriasis lesions, and psoriatic patients treated with ustekinumab and concluded that HSP90 was upregulated in psoriatic lesions and downregulated after ustekinumab treatment [106].

p53 protein plays an important role in cell proliferation control and apoptosis. Baran et al. detected elevated levels of p53 protein in psoriatic skin as compared to nonlesional skin taken from psoriatic patients and skin samples taken from healthy volunteers. The authors concluded that the increased expression of p53 might be the result of the organisms' attempt to counteract proliferation [107].

Ki67 is a marker of cell proliferation and is therefore found in high levels in psoriatic lesions [108]. Some authors showed that Ki67 expression decreases after therapy [109].

Studies support the idea that all the above-mentioned proteins could serve as biomarkers in psoriasis [38].

### 5.3. Other Biomarkers

#### 5.3.1. Markers of Oxidative Stress

Oxidative stress (OS) represents the imbalance between oxidants and antioxidants in the favor of oxidants. The skin is the interface between the human body and the environment and is therefore a major target for oxidative stress. Reactive oxygen species (ROS) can be produced by external factors like smoking, UVR, microorganisms, or air pollution or endogenous factors such as oxidation and reduction reactions from normal cellular metabolism. Endogenous antioxidants like superoxide dismutase (SOD), catalase (CAT), glutathione peroxidase (GP), vitamin C, vitamin E, carotenoids, thiol antioxidants, and so forth normally eliminate or attenuate the effects of ROS. When the antioxidant capacity is overwhelmed, the resulting OS causes oxidative damage of membrane lipids and proteins and DNA [38, 110, 111].

Th1 cytokines and Th2 cytokines involved in the pathogenesis of psoriasis generate ROS. TNF-*α*, for example, activates the pathway of phagocytic nicotine adenine dinucleotide phosphate (NAPDH) oxidases and therefore increases ROS. On the other hand, it reduces antioxidants [112, 113]. The increased production of oxygen metabolites is a known characteristic of psoriasis [38].

Several studies measured markers of oxidative stress in patients with psoriasis. The concentrations of malondialdehyde (MDA), SOD, CAT, total bilirubin, direct bilirubin, uric acid, apolipoproteins, and paraoxonase 1 (PON1) were measured in 100 patients with psoriasis and 100 controls. The authors found significantly higher levels of MDA in patients with psoriasis than in healthy controls. They also found a significant decrease in the activity of SOD, CAT, and PON1, all very important antioxidants [114].

MDA is the final product of lipid peroxidation whose serum levels correlate with the degree of tissue lipid peroxidation. The levels of MDA and nitric oxide (NO) were measured in the plasma of psoriatic patients and healthy controls, and levels of MDA were measured in lesional and nonlesional tissue from psoriatic patients. Significantly higher plasma levels of MDA and NO were found in psoriatic patients as compared to controls. MDA tissue levels were higher in psoriatic lesions than in nonlesional tissues. No correlation was found between tissue levels of MDA and NO and disease activity [115].

Oxidized low-density lipoproteins (Ox-LDL) are also increased in psoriatic tissue, and anti-Ox-LDL antibodies are increased in the plasma of psoriatic patients. These are considered markers of cardiovascular involvement in patients with psoriasis [116].

Urinary biomarkers of oxidative stress were also proposed by some authors. The levels of nitrate, MDA, and 8-hydroxydeoxyguanosine (8-OHdG) were measured in urine samples from psoriatic patients and patients with atopic dermatitis. Nitrate and 8-OHdG urinary levels were found significantly increased in patients with psoriasis. Patients with atopic dermatitis presented increased levels of nitrate. The severity of psoriasis and atopic dermatitis correlated with the nitrate urinary level [117]. Pruritus and chronic idiopathic urticaria have been associated as well with elevated levels of 8-OHdG [118]. Urinary levels of vitamin C and lipoperoxides (thiobarbituric acid reactive substances, TBARS) were also measured in psoriatic patients. The levels of vitamin C were lower in patients with psoriasis than in healthy controls, and, among patients with psoriasis, those with a more severe disease had lower levels than those with psoriasis in remission. The levels of lipoperoxides were higher in psoriatic patients than in controls, the most severely affected patients having the highest levels [119].

Several authors analyzed the effect of different therapies on oxidative stress in patients with psoriasis. The results are conflicting, some authors reporting increased levels of antioxidants after treatment and others reporting no significant changes. Biological therapies such as infliximab and etanercept, however, seem to decrease the levels of ROS and CRP, while increasing the levels of antioxidants [112, 113].

#### 5.3.2. Genetic Markers

Psoriasis is a genetically transmitted disorder. The mode of inheritance is not well understood. The risk of developing psoriasis is 41% in individuals whose both parents are affected and 14% when only one parent is affected. There is also a concordance of psoriasis of up to 73% in monozygotic twins [2]. Psoriasis has been associated with several human leukocyte antigen (HLA) alleles like HLA-Cw6, HLA-B37, HLA-B13, HLA-B57, HLA-Cw1, HLA-DR7, and HLA-DQ9. The strongest relationship is between psoriasis and HLA-Cw6. Patients with this allele are more likely to have early-onset psoriasis. Some authors consider that patients who have a family history of psoriasis, disease onset before the age of 40 years, and expression of HLA-Cw6 have type I psoriasis while patients with no history of the disease, psoriasis onset after the age of 40 years, and lack of HLA expression have type II psoriasis. This classification is however not generally accepted because of the frequent overlap [2, 4].

Genome-wide association studies identified at least 13 major psoriasis susceptibility loci. Psoriasis susceptibility 1 (PSORS1) is the most important susceptibility locus, and it is located on chromosome 6p21. Allen et al. found an association between PSORS1 and early-onset psoriasis, defined as psoriasis occurring before the age of 50 years. According to the authors, identifying the association with PSORS1 might be a better way to discriminate between type I and type II psoriasis [120].

New susceptibility loci for psoriasis have been identified, including RUNX3, TAGAP, and STAT3 involved in regulating T cell function, DDX58, engaged in INF-mediated antiviral responses, ZC3H12C with a role in macrophage activation, and CARD14 and CARM1 involved in NF-*κ*B signaling [99, 121].

Sequence variants in the genes for the IL-23 receptor (IL-23R) and IL-12B have been shown to have a role in the pathogenesis of chronic epithelial inflammation [122].

Other genes associated with psoriasis are genes encoding zinc-finger protein 313 (ZNF313), TNFAIP3 interacting protein 1 (TNIP1), and TNF-*α*-induced protein 3 (TNFAIP3) within the nuclear factor- (NF-) *κ*B pathway [99, 123].

#### 5.3.3. Cell Subsets

Th1, Th17, and Th22 cells all have an important role in the pathogenesis of psoriasis and can be detected in psoriatic lesions. Kagami et al. used flow cytometry to identify and quantify these cells among CD4^+^ circulating cells from patients with psoriasis and found that Th1 cells, Th17 cells, and Th22 cells are increased in the plasma of psoriatic patients. They also found that circulating levels of Th1 and Th17 decreased after infliximab treatment [124].

Circulating natural killer (NK) T cells are found in higher percentages in patients with cutaneous psoriasis than in controls and patients with psoriatic arthritis [35].

Cell subsets were also measured in patients with psoriasis exposed to acute psychosocial stress. In a study performed on 23 patients with psoriasis and 25 healthy controls who were exposed to a standardized laboratory stressor, monocyte and CD4^+^ cell number were significantly higher in psoriatic patients than in controls and CD3^+^/CD5^+^ were significantly decreased in psoriatic patients than in controls. According to the authors, these changes might explain the role of stressors in triggering psoriatic eruption [45].

Despite the promising results, more studies are required to support the use of cell subsets as biomarkers in psoriasis [35].

#### 5.3.4. Cortisol Level

Since more than half of the psoriatic patients declare that the disease is aggravated by psychological stress, some authors evaluated the role of cortisol in the exacerbation of the disorder. One study performed on 40 patients with psoriasis and 40 controls who were exposed to acute psychological stressors found that patients with psoriasis had lower salivary cortisol levels at baseline and lower serum cortisol levels after stress exposure and that the levels were particularly low in those psoriatic patients who declared that stress influenced the evolution of the disease [125]. Other authors examined the influence of daily stressors on cortisol levels in patients with psoriasis and found that high levels of stressors were associated with low cortisol levels and that they were predictors of disease exacerbation [48]. Since other authors did not find a correlation between the level of cortisol and psoriasis flares, further studies are necessary to support the role of HPA axis in psoriasis [46, 126].

## 6. Biomarkers in Psoriatic Arthritis

Studies show that psoriatic arthritis is underdiagnosed in patients with psoriasis vulgaris probably due to the heterogeneity of this condition but also to the lack of systemic biomarkers. Several authors therefore tried to identify biomarkers for psoriatic arthritis which could improve the diagnosis of this disease [35].

A study performed on 52 patients with psoriasis (26 of those with psoriasis alone and 26 with psoriasis and psoriatic arthritis) and 26 controls aimed to identify serum biomarkers for psoriatic arthritis and psoriasis. The authors found higher levels of highly sensitive CRP (hsCRP), osteoprotegerin (OPG), and MMP-3 in patients with psoriasis and psoriatic arthritis as compared to patients with psoriasis alone and concluded that these could be biomarkers for psoriatic arthritis in patients with psoriasis [127]. hsCRP is a sensitive marker of inflammation. OPG is a marker of periostitis, and new bone formation and MMP-3 is an enzyme with a role in bone and cartilage destruction [127].

Other authors showed that the levels of S-calprotectin (S100A8/S100AP) and hsCRP are significantly higher in patients with psoriatic arthritis than in healthy controls. S-Calprotrectin seems to reflect the better burden of the joint disease than hsCRP and is a potential biomarker for psoriatic arthritis [128].

Cytokine levels were also measured in patients with psoriatic arthritis. The levels of IL-1 receptor antagonists (IL-1ra) are correlated with the severity of joint disease [129]. Il-6 is also significantly higher in patients with psoriasis and psoriatic arthritis than patients with psoriasis alone [130].

Synovium and synovial fluid biomarkers were also investigated in patients with psoriatic arthritis. TNF-*α*, IL-6, IFN-*γ*, and IL-1*β* levels are elevated in the synovium of those patients. A connection between psoriatic arthritis and natural killer cell pathway was also described [35].

With regard to genetic biomarkers, HLA-Cw6 is associated with both skin psoriasis and psoriatic arthritis. HLA-B27 is associated with psoriatic arthritis, and it is an indicator of a more severe disease, more often associated with enthesitis, dactylitis, and peripheral and axial joint damage [131]. HLA-B39 is associated with progression in early disease. HLA-B22 is associated with a lower risk of progression [132].

## 7. Conclusions

Psoriasis is a physically, emotionally, and socially invalidating disorder with a great impact on the patients' quality of life which has captured the interest of several authors in the last years. Stress is an important trigger for both the onset of the disease and its subsequent exacerbations; several of the biomarkers studied for the assessment of psoriasis severity have also been identified in stress-related disorders. While an ideal biomarker for psoriasis was not yet found, there is increasing evidence supporting a potential role of VEGF, TGF-*β*1, HBD-2, and IL-18 in evaluating disease severity. Moreover, CRP, platelet P-selectin, TNF-*α*, and IL-6 could help assess both the skin disease and the associated psychopathology. Since biomarkers have the potential to help identify either new key molecules playing important roles in the pathogenesis of the disease, new treatment targets or better markers of disease severity and response to treatment, the quest for ideal markers for psoriasis vulgaris and psoriatic arthritis continues.

## Figures and Tables

**Table 1:** Biomarkers in psoriasis.

Biomarker	Expression	Reference number
*Soluble biomarkers*		
Serum		
CRP	↑	[52, 53]
ESR	↑	[52]
Haptoglobin	↑	[52]
C3, C4 complement proteins	↑	[52]
Fibrinogen	↑	[52]
Soluble P-selectin	↑	[58]
VEGF	↑	[63, 64, 72]
TGF-*β*1	↑	[69–73, 79]
TIMP-1	↑	[73]
MMP-1	↑	[73]
HBD-2	↑	[78]
S100A8/A9	↑	[84, 86]
S100A12	↑	[86]
IFN-*γ*	↑	[87]
TNF-*α*	↑	[87]
IL-6	↑	[87]
IL-8	↑	[87]
IL-12	↑	[87]
IL-18	↑	[73, 87–89]
MDA	↑	[114, 115]
NO	↑	[72, 115]
SOD	↓	[114]
GP	↓	[114]
Anti-Ox-LDL antibodies	↑	[116]
Cell subsets		
Th1	↑	[124]
Th17	↑	[124]
Th22	↑	[124]
*Tissue-associated biomarkers*		
K6, K16	↑	[100, 101]
K1, K10	↓	[100, 101]
Connexin 30	↑	[99, 102]
Connexin 26	↑	[99, 103]
BCL-2	↑	[104]
BCL-X	↑	[104]
Heat-shock proteins	↑	[105, 106]
p53	↑	[107]
Ki67	↑	[108, 109]
Ox-LDL	↑	[116]
MDA	↑	[115]
*Biomarkers for both psychopathology and psoriasis*		
CRP	↑	[52–55]
Soluble P-selectin	↑	[58, 59]
TNF-*α*	↑	[23, 87]
IL-6	↑	[22, 23, 87]

## References

[1] Vachatova S., Andrys C., Krejsek J. (2016). Metabolic syndrome and selective inflammatory markers in psoriatic patients. *Journal of Immunology Research*.

[2] Wolff K., Goldsmith L., Katz S. (2012). *Fitzpatrick’s Dermatology in General Medicine*.

[3] Burns T., Breathnach S., Cox N., Griffiths C. (2010). *Rook’s Textbook of Dermatology*.

[4] Jorizzo J. L., Bolognia J. L., Schaffer J. V. (2012). *Dermatology*.

[5] Hay R. J., Johns N. E., Williams H. C. (2014). The global burden of skin disease in 2010: an analysis of the prevalence and impact of skin conditions. *Journal of Investigative Dermatology*.

[6] Dalgard F. J., Gieler U., Tomas-Aragones L. (2015). The psychological burden of skin diseases: a cross-sectional multicenter study among dermatological out-patients in 13 European countries. *Journal of Investigative Dermatology*.

[7] Pompili M., Innamorati M., Forte A. (2017). Psychiatric comorbidity and suicidal ideation in psoriasis, melanoma and allergic disorders. *International Journal of Psychiatry in Clinical Practice*.

[8] Pompili M., Innamorati M., Trovarelli S. (2016). Suicide risk and psychiatric comorbidity in patients with psoriasis. *Journal of International Medical Research*.

[9] Møller A., Erntoft S., Vinding G., Jemec G. B., Karaoghlanian N. (2015). A systematic literature review to compare quality of life in psoriasis with other chronic diseases using EQ-5D-derived utility values. *Patient Related Outcome Measures*.

[10] Fortune D. G., Richards H. L., Griffiths C. E. (2005). Psychologic factors in psoriasis: consequences, mechanisms, and interventions. *Dermatologic Clinics*.

[11] Kimball A. B., Jacobson C., Weiss S., Vreeland M. G., Wu Y. (2005). The psychosocial burden of psoriasis. *American Journal of Clinical Dermatology*.

[12] Basavaraj K. H., Navya M. A., Rashmi R. (2011). Stress and quality of life in psoriasis: an update. *International Journal of Dermatology*.

[13] JJ W., Penfold R. B., Primatesta P. (2017). The risk of depression, suicidal ideation and suicide attempt in patients with psoriasis, psoriatic arthritis or ankylosing spondylitis. *Journal of the European Academy of Dermatology and Venereology*.

[14] Kurd S. K., Troxel A. B., Crits-Christoph P., Gelfand J. M. (2010). The risk of depression, anxiety and suicidality in patients with psoriasis: a population-based cohort study. *Archives of Dermatology*.

[15] Sarbu M. I., Sarbu A. E., Georgescu S. R. (2014). Sexual dysfunctions in psoriatic patients. *Journal of Mind and Medical Sciences*.

[16] Ferreira B. I., Abreu J. L., Reis J. P., Figueiredo A. M. (2016). Psoriasis and associated psychiatric disorders: a systematic review on etiopathogenesis and clinical correlation. *The Journal of Clinical and Aesthetic Dermatology*.

[17] Gupta M. A., Schork N. J., Gupta A. K., Kirkby S., Ellis C. N. (1993). Suicidal ideation in psoriasis. *International Journal of Dermatology*.

[18] Singh S., Taylor C., Kornmehl H., Armstrong A. W. (2017). Psoriasis and suicidality: a systematic review and meta-analysis. *Journal of the American Academy of Dermatology*.

[19] Egeberg A., Hansen P. R., Gislason G. H., Skov L., Mallbris L. (2016). Risk of self-harm and nonfatal suicide attempts, and completed suicide in patients with psoriasis: a population-based cohort study. *British Journal of Dermatology*.

[20] Fortune D. G., Main C. J., O'Sullivan T. M., Griffiths C. E. (1997). Assessing illness-related stress in psoriasis: the psychometric properties of the Psoriasis Life Stress Inventory. *Journal of Psychosomatic Research*.

[21] Moynihan J., Rieder E., Tausk F. (2010). Psychoneuroimmunology: the example of psoriasis. *Giornale Italiano di Dermatologia e Venereologia*.

[22] Steptoe A., Hamer M., Chida Y. (2007). The effects of acute psychological stress on circulating inflammatory factors in humans: a review and meta-analysis. *Brain, Behavior, and Immunity*.

[23] Maes M., Song C., Lin A. (1998). The effects of psychological stress on humans: increased production of pro-inflammatory cytokines and Th1-like response in stress-induced anxiety. *Cytokine*.

[24] Reichenberg A., Yirmiya R., Schuld A. (2001). Cytokine-associated emotional and cognitive disturbances in humans. *Archives of General Psychiatry*.

[25] Tyring S., Gottlieb A., Papp K. (2006). Etanercept and clinical outcomes, fatigue, and depression in psoriasis: double-blind placebo-controlled randomised phase III trial. *The Lancet*.

[26] Miller A. H., Maletic V., Raison C. L. (2009). Inflammation and its discontents: the role of cytokines in the pathophysiology of major depression. *Biological Psychiatry*.

[27] Raison C. L., Capuron L., Miller A. H. (2006). Cytokines sing the blues: inflammation and the pathogenesis of depression. *Trends in Immunology*.

[28] Finlay A. Y., Khan G. K. (1994). Dermatology Life Quality Index (DLQI)—a simple practical measure for routine clinical use. *Clinical and Experimental Dermatology*.

[29] Sarkar R., Chugh S., Bansal S. (2016). General measures and quality of life issues in psoriasis. *Indian Dermatology Online Journal*.

[30] Gupta M. A., Gupta A. K. (1995). The Psoriasis Life Stress Inventory: a preliminary index of psoriasis-related stress. *Acta Derm Venereol*.

[31] Lewis V. J., Finlay A. Y. (2005). Two decades experience of the Psoriasis Disability Index. *Dermatology*.

[32] McKenna S. P., Cook S. A., Whalley D. (2003). Development of the PSORIQoL, a psoriasis-specific measure of quality of life designed for use in clinical practice and trials. *British Journal of Dermatology*.

[33] Bhosle M. J., Kulkarni A., Feldman S. R., Balkrishnan R. (2006). Quality of life in patients with psoriasis. *Health and Quality of Life Outcomes*.

[34] Langley R. G., Ellis C. N. (2004). Evaluating psoriasis with psoriasis area and severity index, psoriasis global assessment, and lattice system physician’s global assessment. *Journal of the American Academy of Dermatology*.

[35] Molteni S., Reali E. (2012). Biomarkers in the pathogenesis, diagnosis, and treatment of psoriasis. *Psoriasis: Targets and Therapy*.

[36] Colburn W. A., DeGruttola V. G., DeMets D. L. (2001). Biomarkers and surrogate endpoints: preferred definitions and conceptual framework. *Clinical Pharmacology & Therapeutics*.

[37] Ryan C., Kelleher J., Fagan M. F. (2014). Genetic markers of treatment response to tumour necrosis factor-*α* inhibitors in the treatment of psoriasis. *Clinical and Experimental Dermatology*.

[38] Rashmi R., Rao K. S., Basavaraj K. H. (2009). A comprehensive review of biomarkers in psoriasis. *Clinical and Experimental Dermatology*.

[39] Mudigonda P., Mudigonda T., Feneran A. N., Alamdari H. S., Sandoval L., Feldman S. R. (2012). Interleukin-23 and interleukin-17: importance in pathogenesis and therapy of psoriasis. *Dermatology Online Journal*.

[40] Zenewicz L. A., Flavell R. A. (2011). Recent advances in IL-22 biology. *International Immunology*.

[41] Larrick J. W., Morhenn V., Chiang Y. L., Shi T. (1989). Activated Langerhans cells release tumor necrosis factor. *Journal of Leukocyte Biology*.

[42] Schottelius A. J., Moldawer L. L., Dinarello C. A., Asadullah K., Sterry W., Edwards C. K. (2004). Biology of tumor necrosis factor-*α*– implications for psoriasis. *Experimental Dermatology*.

[43] Fitch E., Harper E., Skorcheva I., Kurtz S. E., Blauvelt A. (2007). Pathophysiology of psoriasis: recent advances on IL-23 and Th17 cytokines. *Current Rheumatology Reports*.

[44] FitzGerald O., Winchester R. (2009). Psoriatic arthritis: from pathogenesis to therapy. *Arthritis Research & Therapy*.

[45] Buske-Kirschbaum A., Kern S., Ebrecht M., Hellhammer D. H. (2007). Altered distribution of leukocyte subsets and cytokine production in response to acute psychosocial stress in patients with psoriasis vulgaris. *Brain, Behavior, and Immunity*.

[46] Hall J. M., Podawiltz A., Mummert D. I., Jones H., Mummert M. E. (2012). Psychological stress and the cutaneous immune response: roles of the HPA axis and the sympathetic nervous system in atopic dermatitis and psoriasis. *Dermatology Research and Practice*.

[47] Harvima I. T., Viinamäki H., Naukkarinen A. (1993). Association of cutaneous mast cells and sensory nerves with psychic stress in psoriasis. *Psychotherapy and Psychosomatics*.

[48] Evers A. W., Verhoeven E. W., Kraaimaat F. W. (2010). How stress gets under the skin: cortisol and stress reactivity in psoriasis. *British Journal of Dermatology*.

[49] Connor C. J., Liu V., Fiedorowicz J. G. (2015). Exploring the physiological link between psoriasis and mood disorders. *Dermatology Research and Practice*.

[50] Cemil B. Ç., Canpolat F., Yılmazer D., Eskioğlu F., Alper M. (2012). The association of PASI scores with CRH-R1 expression in patients with psoriasis. *Archives of Dermatological Research*.

[51] Vasiadi M., Therianou A., Alysandratos K. D. (2012). Serum neurotensin (NT) is increased in psoriasis and NT induces vascular endothelial growth factor release from human mast cells. *British Journal of Dermatology*.

[52] Rocha-Pereira P., Santos-Silva A., Rebelo I., Figueiredo A., Quintanilha A., Teixeira F. (2004). The inflammatory response in mild and in severe psoriasis. *British Journal of Dermatology*.

[53] Coimbra S., Oliveira H., Reis F. (2010). C-Reactive protein and leucocyte activation in psoriasis *vulgaris* according to severity and therapy. *Journal of the European Academy of Dermatology and Venereology*.

[54] Gegenava T., Gegenava M., Kavtaradze G. (2011). C-Reactive protein level correlation with depression and anxiety among patients with coronary artery disease. *Georgian Medical News*.

[55] Wium-Andersen M. K., Ørsted D. D., Nielsen S. F., Nordestgaard B. G. (2013). Elevated C-reactive protein levels, psychological distress, and depression in 73 131 individuals. *JAMA Psychiatry*.

[56] Cepeda M. S., Stang P., Makadia R. (2016). Depression is associated with high levels of C-reactive protein and low levels of fractional exhaled nitric oxide: results from the 2007-2012 National Health and Nutrition Examination Surveys. *The Journal of Clinical Psychiatry*.

[57] Koedam J. A., Cramer E. M., Briend E., Furie B., Furie B. C., Wagner D. D. (1992). P-Selectin, a granule membrane protein of platelets and endothelial cells, follows the regulated secretory pathway in AtT-20 cells. *The Journal of Cell Biology*.

[58] Garbaraviciene J., Diehl S., Varwig D. (2010). Platelet P-selectin reflects a state of cutaneous inflammation: possible application to monitor treatment efficacy in psoriasis. *Experimental Dermatology*.

[59] Aschbacher K., Mills P. J., von Känel R. (2008). Effects of depressive and anxious symptoms on norepinephrine and platelet P-selectin responses to acute psychological stress among elderly caregivers. *Brain, Behavior, and Immunity*.

[60] Marina M. E., Roman I. I., Constantin A. M., Mihu C. M., Tătaru A. D. (2015). VEGF involvement in psoriasis. *Clujul Medical*.

[61] Canavese M., Altruda F., Ruzicka T., Schauber J. (2010). Vascular endothelial growth factor (VEGF) in the pathogenesis of psoriasis—a possible target for novel therapies?. *Journal of Dermatological Science*.

[62] Bhushan M., McLaughlin B., Weiss J. B., Griffiths C. E. M. (1999). Levels of endothelial cell stimulating angiogenesis factor and vascular endothelial growth factor are elevated in psoriasis. *British Journal of Dermatology*.

[63] Flisiak I., Zaniewski P., Rogalska-Taranta M., Chodynicka B. (2012). Effect of psoriasis therapy on VEGF and its soluble receptors serum concentrations. *Journal of the European Academy of Dermatology and Venereology*.

[64] Chen H. Q., Li X., Tang R. (2016). Effects of narrow band ultraviolet B on serum levels of vascular endothelial growth factor and interleukin-8 in patients with psoriasis. *American Journal of Therapeutics*.

[65] Akman A., Yilmaz E., Mutlu H., Ozdogan M. (2009). Complete remission of psoriasis following bevacizumab therapy for colon cancer. *Clinical and Experimental Dermatology*.

[66] Schonthaler H. B., Huggenberger R., Wculek S. K., Detmar M., Wagner E. F. (2009). Systemic anti-VEGF treatment strongly reduces skin inflammation in a mouse model of psoriasis. *Proceedings of the National Academy of Sciences of the United States of America*.

[67] Nowacka M., Obuchowicz E. (2013). BDNF and VEGF in the pathogenesis of stress-induced affective diseases: an insight from experimental studies. *Pharmacological Reports*.

[68] Han G., Williams C. A., Salter K., Garl P. J., Li A. G., Wang X. J. (2010). A role for TGF*β* signaling in the pathogenesis of psoriasis. *Journal of Investigative Dermatology*.

[69] Flisiak I., Chodynicka B., Porebski P., Flisiak R. (2002). Association between psoriasis severity and transforming growth factor *β*_1_ and *β*_2_ in plasma and scales from psoriatic lesions. *Cytokine*.

[70] Flisiak I., Porębski P., Flisiak R., Chodynicka B. (2003). Plasma transforming growth factor *β*_1_ as a biomarker of psoriasis activity and treatment efficacy. *Biomarkers*.

[71] Zaher H., Shaker O. G., EL-Komy M. H., El-Tawdi A., Fawzi M., Kadry D. (2009). Serum and tissue expression of transforming growth factor beta 1 in psoriasis. *Journal of the European Academy of Dermatology and Venereology*.

[72] Meki A. R., Al-Shobaili H. (2014). Serum vascular endothelial growth factor, transforming growth factor *β*1, and nitric oxide levels in patients with psoriasis vulgaris: their correlation to disease severity. *Journal of Clinical Laboratory Analysis*.

[73] Flisiak I., Zaniewski P., Chodynicka B. (2008). Plasma TGF-*β*_1_, TIMP-1, MMP-1 and IL-18 as a combined biomarker of psoriasis activity. *Biomarkers*.

[74] You Z., Luo C., Zhang W. (2011). Pro- and anti-inflammatory cytokines expression in rat’s brain and spleen exposed to chronic mild stress: involvement in depression. *Behavioural Brain Research*.

[75] Morizane S., Gallo R. L. (2012). Antimicrobial peptides in the pathogenesis of psoriasis. *The Journal of Dermatology*.

[76] Dongsheng L., Jiawen L., Yiqun D., Xiaoyong Z. (2004). Expression of LL-37, human beta defensin-2, and CCR6 mRNA in patients with psoriasis vulgaris. *Journal of Huazhong University of Science and Technology [Medical Sciences]*.

[77] Hollox E. J., Huffmeier U., Zeeuwen P. L. (2008). Psoriasis is associated with increased *β*-defensin genomic copy number. *Nature Genetics*.

[78] Jansen P. A., Rodijk-Olthuis D., Hollox E. J. (2009). *β*-Defensin-2 protein is a serum biomarker for disease activity in psoriasis and reaches biologically relevant concentrations in lesional skin. *PLoS One*.

[79] Jin T., Sun Z., Chen X. (2017). Serum human beta-defensin-2 is a possible biomarker for monitoring response to JAK inhibitor in psoriasis patients. *Dermatology*.

[80] Kolbinger F., Loesche C., Valentin M. A. (2017). *β*-Defensin 2 is a responsive biomarker of IL-17A–driven skin pathology in patients with psoriasis. *The Journal of Allergy and Clinical Immunology*.

[81] SC H., HS Y., Yen F. L., Lin C. L., Chen G. S., Lan C. C. (2016). Neutrophil extracellular trap formation is increased in psoriasis and induces human *β*-defensin-2 production in epidermal keratinocytes. *Scientific Reports*.

[82] Aberg K. M., Radek K. A., Choi E. H. (2007). Psychological stress downregulates epidermal antimicrobial peptide expression and increases severity of cutaneous infections in mice. *The Journal of Clinical Investigation*.

[83] Martin-Ezquerra G., Mao-Qiang M. A., Melanie H. U. (2011). Psychological stress regulates antimicrobial peptide expression by both glucocorticoid and *β*-adrenergic mechanisms. *European Journal of Dermatology*.

[84] Benoit S., Toksoy A., Ahlmann M. (2006). Elevated serum levels of calcium-binding S100 proteins A8 and A9 reflect disease activity and abnormal differentiation of keratinocytes in psoriasis. *British Journal of Dermatology*.

[85] Anderson K. S., Wong J., Polyak K., Aronzon D., Enerbäck C. (2009). Detection of psoriasin/S100A7 in the sera of patients with psoriasis. *British Journal of Dermatology*.

[86] Wilsmann-Theis D., Wagenpfeil J., Holzinger D. (2016). Among the S100 proteins, S100A12 is the most significant marker for psoriasis disease activity. *Journal of the European Academy of Dermatology and Venereology*.

[87] Arican O., Aral M., Sasmaz S., Ciragil P. (2005). Serum levels of TNF-*α*, IFN-*γ*, IL-6, IL-8, IL-12, IL-17, and IL-18 in patients with active psoriasis and correlation with disease severity. *Mediators of Inflammation*.

[88] Pietrzak A., Lecewicz-Torun B., Chodorowska G., Rolinski J. (2003). Interleukin-18 levels in the plasma of psoriatic patients correlate with the extent of skin lesions and the PASI score. *Acta Dermato-Venereologica*.

[89] Flisiak I., Klepacki A., Chodynicka B. (2006). Plasma and scales levels of interleukin 18 in comparison with other possible clinical and laboratory biomarkers of psoriasis activity. *Biomarkers*.

[90] Mastrolonardo M., Alicino D., Zefferino R., Pasquini P., Picardi A. (2007). Effect of psychological stress on salivary interleukin-1*β* in psoriasis. *Archives of Medical Research*.

[91] Hirotsu C., Rydlewski M., Araujo M. S., Tufik S., Andersen M. L. (2012). Sleep loss and cytokines levels in an experimental model of psoriasis. *PLoS One*.

[92] Schick C., Kamachi Y., Bartuski A. J. (1997). Squamous cell carcinoma antigen 2 is a novel serpin that inhibits the chymotrypsin-like proteinases cathepsin G and mast cell chymase. *Journal of Biological Chemistry*.

[93] Chechlinska M., Kowalewska M., Brzoska-Wojtowicz E. (2010). Squamous cell carcinoma antigen 1 and 2 expression in cultured normal peripheral blood mononuclear cells and in vulvar squamous cell carcinoma. *Tumor Biology*.

[94] Piruzian E. S., Sobolev V. V., Abdeev R. M. (2009). Study of molecular mechanisms involved in the pathogenesis of immune-mediated inflammatory diseases, using psoriasis as a model. *Acta Naturae*.

[95] Watanabe Y., Yamaguchi Y., Komitsu N. (2016). Elevation of serum squamous cell carcinoma antigen 2 in patients with psoriasis: associations with disease severity and response to the treatment. *British Journal of Dermatology*.

[96] Sano T., Kume T., Fujimura T. (2009). Long-term alteration in the expression of keratins 6 and 16 in the epidermis of mice after chronic UVB exposure. *Archives of Dermatological Research*.

[97] Komine M., Rao L. S., Freedberg I. M., Simon M., Milisavljevic V., Blumenberg M. (2001). Interleukin-1 induces transcription of keratin K6 in human epidermal keratinocytes. *Journal of Investigative Dermatology*.

[98] Mommers J. M., Van Rossum M. M., Van Erp P. E., Van De Kerkhof P. C. (2000). Changes in keratin 6 and keratin 10 (co-) expression in lesional and symptomless skin of spreading psoriasis. *Dermatology*.

[99] Jiang S., Hinchliffe T. E., Wu T. (2015). Biomarkers of an autoimmune skin disease—psoriasis. *Genomics, Proteomics & Bioinformatics*.

[100] Bhawan J., Bansal C., Whren K., Schwertschlag U. (2004). K16 expression in uninvolved psoriatic skin: a possible marker of pre-clinical psoriasis. *Journal of Cutaneous Pathology*.

[101] Franssen M. E., Boezeman J. B., van de Kerkhof P. C., van Erp P. E. (2004). Monitoring hyperproliferative disorders in human skin: flow cytometry of changing cytokeratin expression. *Cytometry Part B: Clinical Cytometry*.

[102] Lemaitre G., Sivan V., Lamartine J. (2006). Connexin 30, a new marker of hyperproliferative epidermis. *British Journal of Dermatology*.

[103] Lucke T., Choudhry R., Thom R., Selmer I. S., Burden A. D., Hodgins M. B. (1999). Upregulation of connexin 26 is a feature of keratinocyte differentiation in hyperproliferative epidermis, vaginal epithelium, and buccal epithelium. *Journal of Investigative Dermatology*.

[104] Koçak M., Bozdoǧan Ö., Erkek E., Atasoy P., Birol A. (2003). Examination of Bcl-2, Bcl-X and bax protein expression in psoriasis. *International Journal of Dermatology*.

[105] Beere H. M. (2004). ‘The stress of dying’: the role of heat shock proteins in the regulation of apoptosis. *Journal of Cell Science*.

[106] Kakeda M., Arock M., Schlapbach C., Yawalkar N. (2014). Increased expression of heat shock protein 90 in keratinocytes and mast cells in patients with psoriasis. *Journal of the American Academy of Dermatology*.

[107] Baran W., Szepietowski J. C., Szybejko-Machaj G. (2005). Expression of p53 protein in psoriasis. *Acta Dermatovenerologica Alpina Panonica et Adriatica*.

[108] Abdou A. G., Maraee A. H., Eltahmoudy M., El-Aziz R. A. (2013). Immunohistochemical expression of GLUT-1 and Ki-67 in chronic plaque psoriasis. *The American Journal of Dermatopathology*.

[109] Jesionek-Kupnicka D. O., Chomiczewska-Skóra D. O., Rotsztejn H. E. (2013). Influence of phototherapy in psoriasis on Ki-67 antigen expression: a preliminary study. *Polish Journal of Pathology*.

[110] Emre S., Metin A., Demirseren D. D., Kilic S., Isikoglu S., Erel O. (2013). The relationship between oxidative stress, smoking and the clinical severity of psoriasis. *Journal of the European Academy of Dermatology and Venereology*.

[111] Zhou Q., Mrowietz U., Rostami-Yazdi M. (2009). Oxidative stress in the pathogenesis of psoriasis. *Free Radical Biology & Medicine*.

[112] Peluso I., Cavaliere A., Palmery M. (2016). Plasma total antioxidant capacity and peroxidation biomarkers in psoriasis. *Journal of Biomedical Science*.

[113] Bacchetti T., Campanati A., Ferretti G., Simonetti O., Liberati G., Offidani A. M. (2013). Oxidative stress and psoriasis: the effect of antitumour necrosis factor-*α* inhibitor treatment. *British Journal of Dermatology*.

[114] Houshang N., Reza K., Masoud S., Ali E., Mansour R., Vaisi-Raygani A. (2014). Antioxidant status in patients with psoriasis. *Cell Biochemistry & Function*.

[115] Şikar Aktürk A., Özdoğan H. K., Bayramgürler D., Çekmen M. B., Bilen N., Kıran R. (2012). Nitric oxide and malondialdehyde levels in plasma and tissue of psoriasis patients. *Journal of the European Academy of Dermatology and Venereology*.

[116] Gerdes S., Osadtschy S., Buhles N., Baurecht H., Mrowietz U. (2014). Cardiovascular biomarkers in patients with psoriasis. *Experimental Dermatology*.

[117] Nakai K., Yoneda K., Maeda R. (2009). Urinary biomarker of oxidative stress in patients with psoriasis vulgaris and atopic dermatitis. *Journal of the European Academy of Dermatology and Venereology*.

[118] Dinu L. U., Ene C. D., Nicolae I. L., Tampa M., Matei C., Georgescu S. R. (2014). The serum levels of 8-hidroxy-deoxyguanosine under the chemicals influence. *Revista de Chimie*.

[119] Tampa M., Nicolae I. L., Ene C. D., Sarbu I., Matei C., Georgescu S. R. (2017). Vitamin C and thiobarbituric acid reactive substances (TBARS) in psoriasis vulgaris related to psoriasis area severity index (PASI). *Revista de Chimie*.

[120] Allen M. H., Ameen H., Veal C. (2005). The major psoriasis susceptibility locus PSORS1 is not a risk factor for late-onset psoriasis. *Journal of Investigative Dermatology*.

[121] Tsoi L. C., Spain S. L., Knight J. (2012). Identification of 15 new psoriasis susceptibility loci highlights the role of innate immunity. *Nature Genetics*.

[122] Capon F., Di Meglio P., Szaub J. (2007). Sequence variants in the genes for the interleukin-23 receptor (*IL23R*) and its ligand (*IL12B*) confer protection against psoriasis. *Human Genetics*.

[123] Hüffmeier U., Uebe S., Ekici A. B. (2010). Common variants at *TRAF3IP2* are associated with susceptibility to psoriatic arthritis and psoriasis. *Nature Genetics*.

[124] Kagami S., Rizzo H. L., Lee J. J., Koguchi Y., Blauvelt A. (2010). Circulating Th17, Th22, and Th1 cells are increased in psoriasis. *Journal of Investigative Dermatology*.

[125] Richards H. L., Ray D. W., Kirby B. (2005). Response of the hypothalamic–pituitary–adrenal axis to psychological stress in patients with psoriasis. *British Journal of Dermatology*.

[126] Brouwer S. J., Middendorp H. V., Stormink C. (2014). The psychophysiological stress response in psoriasis and rheumatoid arthritis. *British Journal of Dermatology*.

[127] Chandran V., Cook R. J., Edwin J. (2010). Soluble biomarkers differentiate patients with psoriatic arthritis from those with psoriasis without arthritis. *Rheumatology*.

[128] Hansson C., Eriksson C., Alenius G. M. (2014). S-Calprotectin (S100A8/S100A9): a potential marker of inflammation in patients with psoriatic arthritis. *Journal of Immunology Research*.

[129] Elkayam O., Yaron I., Shirazi I., Yaron M., Caspi D. (2000). Serum levels of IL-10, IL-6, IL-1ra, and sIL-2R in patients with psoriatic arthritis. *Rheumatology International*.

[130] Alenius G. M., Eriksson C., Rantapää Dahlqvist S. (2009). Interleukin-6 and soluble interleukin-2 receptor alpha – markers of inflammation in patients with psoriatic arthritis?. *Clinical and Experimental Rheumatology*.

[131] Queiro R., Morante I., Cabezas I., Acasuso B. (2016). HLA-B27 and psoriatic disease: a modern view of an old relationship. *Rheumatology*.

[132] Gladman D. D., Farewell V. T., Kopciuk K. A., Cook R. J. (1998). HLA markers and progression in psoriatic arthritis. *The Journal of Rheumatology*.

